# The Influence of Transcranial Alternating Current Stimulation on Fatigue Resistance

**DOI:** 10.3390/brainsci13081225

**Published:** 2023-08-21

**Authors:** Kayla A. De Guzman, Richard J. Young, Valentino Contini, Eliza Clinton, Ashley Hitchcock, Zachary A. Riley, Brach Poston

**Affiliations:** 1 Department of Kinesiology and Nutrition Sciences, University of Nevada-Las Vegas, Las Vegas, NV 89154, USA; deguzk1@unlv.nevada.edu (K.A.D.G.); clinte1@unlv.nevada.edu (E.C.);; 2 Optum Labs, Minnetonka, MN 55343, USA; 3 Department of Kinesiology, Indiana University—Purdue University Indianapolis, Indianapolis, IN 46202, USA

**Keywords:** fatigue, muscle, tACS

## Abstract

Previous research has shown that some forms of non-invasive brain stimulation can increase fatigue resistance. The purpose of this study is to determine the influence of transcranial alternating current stimulation (tACS) on the time to task failure (TTF) of a precision grip task. The study utilized a randomized, double-blind, SHAM-controlled, within-subjects design. Twenty-six young adults completed two experimental sessions (tACS and SHAM) with a 7-day washout period between sessions. Each session involved a fatiguing isometric contraction of the right hand with a precision grip with either a tACS or SHAM stimulation applied to the primary motor cortex (M1) simultaneously. For the fatiguing contraction, the participants matched an isometric target force of 20% of the maximum voluntary contraction (MVC) force until task failure. Pre- and post-MVCs were performed to quantify the force decline due to fatigue. Accordingly, the dependent variables were the TTF and MVC force decline as well as the average EMG activity, force error, and standard deviation (SD) of force during the fatiguing contractions. The results indicate that there were no significant differences in any of the dependent variables between the tACS and SHAM conditions (*p* value range: 0.256–0.820). These findings suggest that tACS does not increase the TTF during fatiguing contractions in young adults.

## 1. Introduction

Fatigue is defined as a progressive exercise-induced reduction in the maximal voluntary muscle force-generating capacity [1,2,3,4,5]. Fatigue originates from physiological changes that occur distal to the neuromuscular junction at the level of the muscle (peripheral fatigue) and from changes within the spinal cord, brain stem, and cortex (central fatigue) [1,2,3,4,5]. Due to the importance of muscle fatigue in human performance, extensive research has been performed to uncover the behavioral and physiological adjustments that occur during fatiguing contractions and how these adjustments vary with the details of the motor task [4,5]. These studies have consistently shown that fatigue leads to increases in force variability and decreases in movement accuracy. In addition, a number of interrelated physiological modifications occur during the progression of fatigue such as increases in descending drive from the primary motor cortex (M1) and motor unit recruitment to maintain the required force level [1,2,4,5]. Furthermore, there are decreases in the motor unit discharge rates in some of the active motor units [4,5], increases in the motor unit discharge rate variability [5], decreases in the excitatory Ia afferent input to motor neurons [5], and increases in the afferent feedback to spinal and cortical areas from inhibitory groups III and IV [2,3]. The patterns of intermuscular coordination can also significantly change during fatigue in further attempts to maintain the requisite performance or force levels [6]. Despite these advancements in the study of fatigue, few methods exist to significantly increase fatigue resistance beyond traditional methods of physical training under fatiguing conditions with the application of progressive overload or various well-known nutritional [7] and dietary supplement strategies [8,9]. Thus, the development of effective and practical adjuncts to these traditional approaches would have significant benefits given the role of fatigue in human performance in healthy adults and in various motor disorders.

Transcranial direct current stimulation (tDCS) is the most widely used method of non-invasive electrical brain stimulation with the aim of increasing motor performance [10,11,12,13,14,15]. Most tDCS studies involved motor skill training and have shown that a single 10–20 min application of anodal tDCS to M1 can enhance cortical excitability and increase motor skill by approximately 10–15% compared to practice alone (SHAM stimulation) [10]. In addition, many studies have also shown that tDCS can also mitigate muscle fatigue and increase the time to task failure (TTF) of sustained isometric contractions [16,17,18,19] or the endurance time of several other types of motor tasks [17,20,21,22,23]. For example, in one of the earliest studies on the topic, Cogiamanian and colleagues [16] reported that anodal tDCS applied to M1 increased fatigue resistance by approximately 18% compared to SHAM stimulation in a repeated submaximal isometric elbow flexor paradigm. Anodal tDCS was shown to improve endurance times in cycling in a few other studies [20,21]. Accordingly, three review articles [17,22,23] concluded that the balance of the literature indicates that tDCS can improve fatigue resistance in a variety of motor tasks, although the effects could be viewed as small to moderate [17,22]. Therefore, there is likely room to improve the efficacy of non-invasive electrical brain stimulation methods for fatigue resistance, as only a fraction of the possible forms and parameters of stimulation have been investigated.

Recently, another form of non-invasive electrical brain stimulation termed transcranial alternating current stimulation (tACS) was developed and is being increasingly studied to improve human motor performance [24,25,26,27]. tACS has some characteristics and methodological considerations that are similar to tDCS such as the electrode montages, stimulation durations, targeted brain areas, and current strengths utilized [24,25,26,27]. Accordingly, tACS was shown to increase cortical excitability and enhance motor skill in a similar manner to tDCS when delivered to M1 [25], the cerebellum [26,28], or both areas at the same time [29,30,31]. However, tACS also has a few unique properties that can mediate the increases in cortical excitability [24] and motor performance via some different physiological mechanisms compared with tDCS [24,25,27,32]. Most notably, tACS has the potential to elicit entrainment at specific frequencies of populations of neurons within or between brain regions [24,25,27,32]. This is important, as the synchronization of neuronal activity is a basic mechanism of functional communication at both of these levels [27,32]. Thus, it is theoretically possible that tACS can elicit equal or even greater positive effects on various measures of motor performance compared to tDCS [24,27]. However, the currently available tACS studies only investigated motor skill performance, and the effects of tACS application on motor system fatigue have yet to be examined.

The purpose of this study is to determine the influence of transcranial alternating current stimulation (tACS) on the time to task failure (TTF) of a precision grip task in young adults. The participants performed fatiguing contractions in a tACS condition and a SHAM condition in a crossover design with a week-long washout period. Based on previous M1 tDCS studies that improved muscle fatigue resistance [16,17,18,19,22,23] and provided evidence that tACS can increase cortical excitability [24] and motor learning [25] as well as offer other advantages compared to tDCS [24,27], it was hypothesized that applying tACS to M1 would increase the TTF of a fatiguing contraction involving hand muscles. It was also expected that tACS would lead to a slower rate of rise in EMG activity throughout the fatiguing contraction compared to a SHAM stimulation. In addition, it was predicted that tACS would decrease the decline in the MVC force after the fatiguing contraction as well as decrease the force error and SD of force (force variability) observed during the fatiguing contraction.

## 2. Materials and Methods

### 2.1. Participants

A total of 26 young adults (17 males, 9 females; mean age: 26.4 ± 4.6 years; age range: 18–34 years) participated in the study and provided written informed consent. All subjects were right-handed according to the Edinburgh Handedness Inventory [33], free of any neurological disorders and uncontrolled medical conditions, and did not meet any of the international non-invasive brain stimulation exclusion criteria. This study was approved by the University of Nevada Las Vegas Institutional Review Board (UNLV-2022-422), and all procedures were conducted according to the Declaration of Helsinki.

### 2.2. Experimental Design

This study utilized a randomized, double-blind, SHAM-controlled, within-subjects crossover design. The within-subjects design was chosen for two interrelated reasons. First, the substantial interindividual differences in the responsiveness to non-invasive brain stimulation due to physiological, biological, genetic, and anatomical factors are substantially mitigated [34,35]. Second, the within-subjects design allows for greater statistical power compared with a between-subjects design [36].

### 2.3. Experimental Procedures

**Figure 1:**
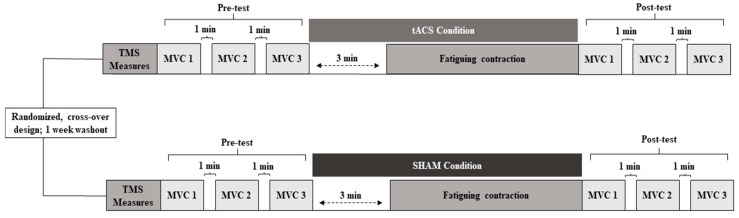
Schematic of experimental design and experimental schedule.

Each participant completed two experimental sessions (tACS and SHAM) with a 7-day washout period between sessions. Participants were randomized into either the tACS or SHAM condition (Research Randomizer; www.randomizer.org; accessed on 15 February 2023). Thus, 13 participants completed the tACS condition first and the SHAM condition second, whereas the other 13 participants completed the conditions in the opposite order. In the first experimental session, participants completed the informed consent form and the Edinburgh Handedness Inventory. Subsequently, each experiment was performed in the following order: (1) transcranial magnetic stimulation (TMS) measurements were undertaken to find the first dorsal interosseus muscle’s motor “hotspot” and determine the resting motor threshold (RMT); (2) pre-MVCs were completed; (3) tACS or SHAM stimulation was applied for 3 min prior and during the entire performance of the fatiguing contraction; and (4) post-MVCs were completed. Thus, the two experimental sessions were identical with the exception of the type of stimulation applied. A schematic of the experimental design and experimental schedule is depicted in Figure 1.

#### 2.3.1. TMS Measures

Surface EMG electrodes were placed on the FDI muscle for TMS testing to measure motor evoked potentials (MEPS) in response to TMS. The motor hotspot of the FDI muscle of the right was located using single-pulse TMS via a Magstim 2002 connected to a double 70 mm remote control figure-of-eight coil [37]. The coil was oriented against the scalp of the left M1 with the handle laterally positioned 45 degrees from the midline over the area of M1 representing the hand. Participants received approximately 20–40 pulses to identify the scalp area that corresponded with the FDI motor hotspot, and this area was marked with a temporary marker for subsequent determination of RMT and tACS electrode placement. Next, RMT of the FDI was measured for each participant and was defined as the lowest TMS intensity as a percentage of maximum stimulator output (% MSO) that induced at least a 50-microvolt peak-to-peak amplitude MEP in five of ten consecutive TMS trials. RMT was measured because it is a basic measure of cortical excitability, and some studies have shown that a lower RMT and related measurement values are associated with greater susceptibility to tDCS compared to individuals with higher values [38,39].

#### 2.3.2. MVCs

The MVCs were performed using a methodology similar to those of prior studies [40,41,42]. In brief, the participants were seated with a small table situated by their right side. The table had a grip manipulandum instrumented with two force transducers. Participants exerted force on the force transducers with the index finger and thumb of the right hand using a precision grip. The arm was abducted to ~45°, the elbow was flexed to ~90°, and the hand was in a semi-supinated position. For each MVC, participants were instructed to create maximum force in the shortest time possible and to hold the maximum force for ~5 s [40,41]. Visual feedback was provided in the form of the total force (sum of index finger and thumb forces) on a computer monitor located on a table in front of the participants. 

A total of 3 trials were performed both before and immediately after the stimulation period and fatiguing contraction (Figure 1) with one minute of rest between trials. The MVC trial performed before the fatiguing contraction that exhibited the highest force was denoted as pre-MVC and was the reference value to calculate the target force for the subsequent fatiguing contraction for each participant. Conversely, the first MVC performed immediately after the fatiguing contraction was denoted as post-MVC and was used to calculate the percentage decline in the MVC force relative to the pre-MVC force to quantify fatigue. Note that post-MVC was performed almost immediately after the fatiguing contraction (~10–15 s) as it was performed as quickly as the experimenter could reset the computer following task failure to collect MVCs. Finally, the last two MVCs were performed with one minute of rest between trials.

#### 2.3.3. tACS Application and Electrode Placement

High-frequency tACS (70 Hz) was delivered at a current strength of 1 mA through two rubber electrodes (5 × 7 cm) covered by sponges soaked in saline using a NeuroConn DC Stimulator Plus/MR. The target electrode was placed over the earlier identified FDI motor hotspot of the left M1, and the reference electrode was placed on the contralateral supraorbital region. This electrode montage and set of tACS stimulation parameters were chosen for three interrelated reasons based on a study [25] by Sugata et al. (2018) and a series of similar studies. First, Sugata et al. (2018) found that those parameters elicited significant oscillatory neural activity and enhanced motor learning [25]. Second, the study also had concurrent magnetoencephalography recordings that confirmed a functional relationship between the oscillatory neural activity and motor learning. Third, several other tACS studies found improvements in motor skill using tACS applied at 70 Hz, albeit these studies applied tACS over M1 and the cerebellum concurrently [29,30,31]. For the SHAM condition, the same tACS parameters were applied, but only for a total of one minute of stimulation time. 

Importantly, the duration of stimulation varied in the tACS condition depending on the participant’s time to task failure, but was no longer than 20 min. Specifically, the stimulator ran for 3 min prior [43] to starting the fatiguing contraction and was kept on until task failure (Figure 1), which resulted in slightly different tACS application times due to the range in TTF values across the participants. The stimulator was operated by an investigator who did not take part in data collection, and the investigators who managed the experiments were blind to the experimental conditions as in previous studies [37,40,41].

#### 2.3.4. Fatiguing Contraction

The fatiguing contraction task was performed using the same experimental arrangement and hand positioning as the precision grip task used in previous motor skill studies [40,41], and utilized the same hand posture as used in the MVC task. The overall methodology utilized for the fatiguing contraction task was also similar to a previous study [6]. Participants were instructed to accomplish a sustained isometric contraction for as long as possible until failure at a target force of 20% of the pre-MVC. Visual feedback of the target force was given on a monitor in the form of a black horizontal line placed in the middle of the screen. Accordingly, the total force produced by the index finger and thumb was superimposed on the screen in the form of a red trace. Thus, participants could see the force they produced relative to the target line in real time and were directed to match their force trace to the target force as precisely as possible for the time of the fatiguing contraction. The duration that the fatiguing contraction task was sustained was denoted as the TTF. The criteria of termination [6] for the fatiguing contraction included (1) the inability to sustain the force exerted within 10% of the target force for 3 s; (2) the failure to maintain the same hand or forearm posture during the trial; and (3) the inability to sustain the target force (participant gave up and allowed the force to completely drop). However, all participants except one failed due to the inability to sustain the target force [6].

#### 2.3.5. Data Analysis

The data were collected in custom-written scripts in Signal software version 5.04 (CED, Cambridge, UK), whereas data were analyzed offline in both custom Signal scripts and using the Python programming language (Fredericksburg, VA, USA). The dependent variables were RMT, pre-MVC, target force, TTF, decline in MVC, average EMG, average force, force error, and SD of force. RMT, pre-MVC, target force, and average force were viewed as control variables as significant differences across those variables between the two conditions performed on each of the two days could be viewed as potential confounding factors. In contrast, the TTF and the decline in MVC were the primary outcome measures, whereas the average EMG, force error, and SD of force were considered secondary outcome measures. Note that the average EMG, force error, and SD of force were calculated both over the entirety of the fatiguing contraction and over four time quartiles (Q1, Q2, Q3, and Q4) of the fatiguing contraction, which were calculated as 0–25%, 26–50%, 51–75%, and 76–100% of the fatiguing contraction time for each participant. This was executed to determine the magnitude and rate of change in these variables over the course of the fatiguing contraction for the two conditions.

The RMT was calculated as the lowest TMS intensity (% MSO) that induced 50-microvolt peak-to-peak amplitude MEPs in five of ten consecutive TMS trials. As mentioned previously, pre-MVC was defined as the maximum of the three MVCs performed before the fatiguing contraction and stimulation, whereas the target force was set as 20% of the pre-MVC in each experimental session. Similarly, the TTF was denoted as the total time in seconds that the fatiguing contraction was sustained. The percentage decline in the MVC was quantified as the difference between the pre-MVC and the first MVC performed after the fatiguing contraction. Therefore, by definition, this decline in force was used as the index of fatigue in each experimental session [1]. The average EMG was determined as a percentage of the highest average rectified EMG recorded during the plateau phase (~5 s) of the MVCs performed before the fatiguing contraction (normalized EMG). This calculation for the average EMG was performed both over the entire fatiguing contraction and for the four time quartiles. 

The average force was calculated as the average force delivered by each participant over the fatiguing contraction. The force error was quantified in a comparable manner to prior motor skill studies [40,41], but in this case, it was quantified as the average error in force relative to the horizontal target force line over the entire course of the fatiguing contraction. More specifically, the absolute value of the difference at each sampling point between the target force line and the force produced by that participant was quantified and then averaged over the fatiguing contraction as well as separately in each time quartile. Finally, the SD of force was simply calculated as the SD of the total force produced either over the entirety of the fatiguing contractions or over each time quartile.

#### 2.3.6. Statistical Analysis

The dependent variables of RMT, pre-MVCs, target force, TTF, and percentage decline in the MVC between the tACS and SHAM conditions were all compared using two-tailed paired *t*-tests. Similarly, the average EMG, average force, force error, and SD of force calculated over the entirety of the fatiguing contraction for the tACS and SHAM conditions were also all compared using two-tailed paired *t*-tests. In contrast, the average EMG, force error, and SD of force that were calculated for each time quartile of the fatiguing contractions were analyzed using two-factor repeated measures ANOVAs: two conditions (tACS and SHAM) × four quartiles (Q1, Q2, Q3, and Q4) with both factors being within-subjects. A significance level for all statistical tests was set at *p* < 0.05. The data are reported as means +/− standard errors in the figures. The effect sizes are reported as Cohen’s d for the *t*-tests and as partial eta squared values for the ANOVAs. Finally, interim futility analyses were conducted similarly to a previous study [40]. The primary dependent variables of the TTF and percentage decline in the MVC were evaluated to determine if additional resources and recruitment were needed. Using the means, standard deviations, and test statistics from these analyses and the “Conditional Power and Sample Size Reestimation of Paired *T*-Tests” module in PASS 20.0.10 (NCSS, LLC. Kaysville, UT, USA), the number of participants was determined to achieve sufficient power to find statistically significant differences. For the TTF, 969 participants would be needed, whereas for the percentage decline in MVC, a total of 332 participants would be needed. Thus, the futility analysis indicated that it was highly improbable that the lack of significant differences between the tACS and SHAM conditions were due to the sample size of the current study. Due to these estimates and the obvious impracticality of recruiting these numbers of participants, we chose to stop the recruitment of additional participants for futility as there was a clear lack of meaningful treatment effects. 

## 3. Results

### 3.1. RMT, Pre-MVC, and Target Force

**Figure 2:**
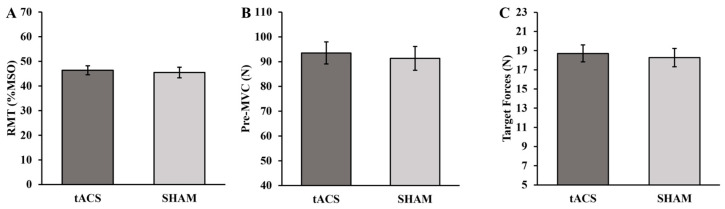
(**A**) Resting motor threshold (RMT), (**B**) pre-maximum voluntary contraction (MVC), and (**C**) target force for the tACS and SHAM conditions.

The RMT was not statistically different for the tACS and SHAM conditions (*p* = 0.395; d = 0.17; Figure 2A). In addition, the pre-MVCs were similar between the conditions (*p* = 0.462; d = 0.147; Figure 2B), and therefore, the target forces were also not significantly different (*p* = 0.460; d = 0.147; Figure 2C) between the tACS and SHAM conditions. 

### 3.2. TTF, Decline in MVC Force, and EMG Activity

**Figure 3:**
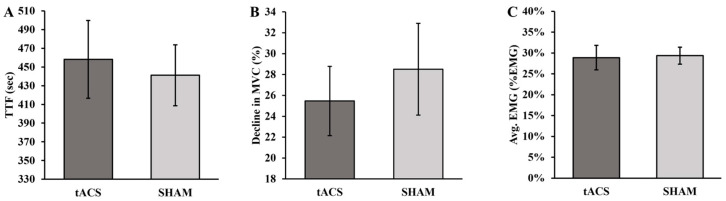
(**A**) Time to task failure (TTF), (**B**) decline in maximum voluntary contraction (MVC) force, and (**C**) electromyographic (EMG) activity for the tACS and SHAM conditions.

The TTF was not significantly different between the tACS and SHAM conditions (*p* = 0.503; d = 0.133; Figure 3A). Similarly, the percentage decline in the MVC between the pre- and post-tests was not statistically different for the tACS and SHAM conditions (*p* = 0.551; d = 0.119; Figure 3B). Accordingly, the average EMG activity for the entirety of the fatiguing contractions was comparable between the tACS and SHAM conditions (*p* = 0.820; d = −0.045; Figure 3C).

### 3.3. Average Force, Force Error, and SD of Force

**Figure 4:**
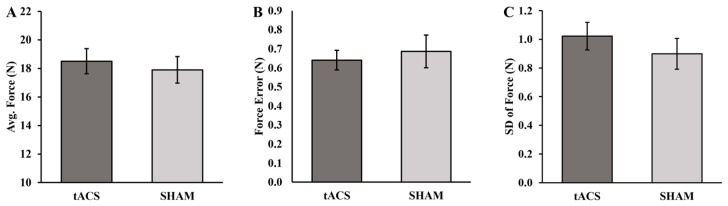
(**A**) Average force, (**B**) force error, and (**C**) standard deviation (SD) of force for the tACS and SHAM conditions.

The average force (*p* = 0.299; d = 0.208), force error (*p* = 0.562; d = −0.115), and SD of force (*p* = 0.256; d = 0.228) over the entire course of the fatiguing contractions were all not significantly different between the tACS and SHAM conditions (Figure 4A–C).

### 3.4. Changes in EMG Activity, Force Error, and SD of Force with Time during Fatigue

**Figure 5:**
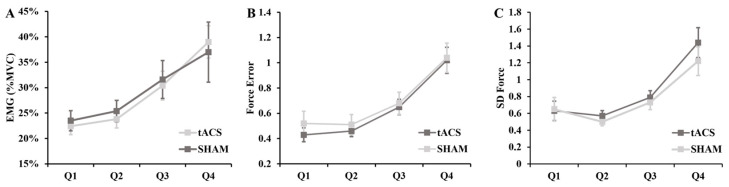
(**A**) Electromyographic (EMG) activity, (**B**) force error, and (**C**) standard deviation (SD) of force changes during fatigue for the tACS and SHAM conditions.

For the EMG activity, there was a main effect for the quartile (*p* < 0.001; η^2^ = 0.395; Figure 5A) as the EMG activity increased progressively during the fatiguing contractions. However, both the main effects for the condition (*p* = 0.820; η^2^ = 0.002) and the condition × quartile interaction were not significant (*p* = 0.392; η^2^ = 0.032). The force error also progressively increased with time during the fatiguing contractions (quartile main effect: *p* < 0.001; η^2^ = 0.556; Figure 5B). In contrast, both the main effects for the condition (*p* = 0.562; η^2^ = 0.014) and condition × quartile interaction were not significant (*p* = 0.668; η^2^ = 0.016). Finally, the SD of force also significantly increased over the course of the fatiguing contractions (quartile main effect: *p* < 0.001; η^2^ = 0.478; Figure 5C). Nonetheless, both the main effects for the condition (*p* = 0.258; η^2^ = 0.051) and condition × quartile interaction were not significant (*p* = 0.024).

## 4. Discussion

The purpose of this study was to determine the influence of transcranial alternating current stimulation (tACS) on the TTF of a precision grip task in young adults. The study produced three main findings: (1) the TTF and the percentage decline in MVC force were similar for the tACS and SHAM conditions; (2) the average EMG activity and increase in the EMG activity over time during the fatiguing contractions were also similar for the tACS and SHAM conditions; and (3) the force error and SD of force significantly increased over the course of the fatiguing contractions, but were not significantly different between the tACS and SHAM conditions. Collectively, these findings imply that a single session of tACS stimulation applied to M1 does not improve fatigue resistance in a precision grip task in young adults.

### 4.1. Influence of tACS on TTF and Decline in MVC

The current study appears to be the first to directly investigate the influence of tACS applied to M1 on fatigue resistance. The TTF and decline in MVC force between the pre- and post-MVC measurements are the most common indices used to quantify the magnitude of fatigue experienced due to sustained, submaximal isometric fatiguing contractions [1,2,3,5]. Accordingly, the current study compared fatigue resistance when the same fatiguing task was performed simultaneously with the administration of tACS versus a SHAM stimulation. It was initially hypothesized that tACS would improve the TTF of the fatigue task to a greater degree than when performing the fatigue task alone in the SHAM condition. Contrary to this set of hypotheses, the TTF and percentage decline in the MVC force between the pre- and post-MVCs were almost identical between the tACS and SHAM conditions. Therefore, tACS did not significantly enhance the fatigue resistance exhibited either during the course of the fatiguing contraction or immediately after task failure. In addition, the current findings could not have been due to potentially confounding influences such as differences in the RMT (baseline cortical excitability), pre-MVCs and the resulting target forces (lower target forces could lead to longer TTF), and the average force produced during the fatiguing contractions as the values for these variables were all not statistically different between the tACS and SHAM conditions performed in the two separate experimental sessions. Therefore, the lack of differences in this set of outcomes should have allowed for the ability to identify differences in the measures of fatigue resistance between the tACS and SHAM conditions if they would have been present.

These negative outcomes conflict with the positive outcomes reported in the majority of prior fatigue studies using single-session tDCS with young adults [16,17,18,19,20,21,22,23]. Although there are currently no tACS studies that directly investigated motor system fatigue in young adults, the present results are also inconsistent with the balance of studies that found improved motor skill acquisition when tACS was applied to M1 [25], the cerebellum [26,28], or both areas simultaneously [29,30,31]. Accordingly, the current observations would seem to support some of the conclusions of both tDCS skill [10] and fatigue review articles [17,22,23] that either a non-trivial minority of studies show no positive effects due to stimulation [44] or that when present, the effects should be considered small to moderate [22], especially in fatigue-related studies. Taken together, these lines of reasoning and the present findings imply that targeting M1 with tACS may not be the most efficacious strategy or non-invasive brain stimulation method to enhance fatigue resistance in healthy young adults.

### 4.2. EMG and Force Change during the Fatiguing Contractions

A consistent set of findings across all submaximal isometric fatiguing contraction studies is that EMG activity and force variability increase substantially over time during the contraction [1,2,3,4]. Accordingly, the average EMG activity, force error, and SD of force increased progressively during both the tACS and SHAM conditions as expected. However, it was also originally hypothesized that tACS would lead to a lower rate of rise in the EMG activity, force error, and SD of force. This was based on the rationale that if tACS could increase motor skill learning as in previous studies, then this mechanism could translate increased proficiency in accurately matching the target force line. Therefore, this would lead to increased efficiency (lower metabolic demands) in performing the current task (e.g., lower magnitudes of force fluctuations) under fatiguing conditions, especially at the beginning of the contraction. Accordingly, the resulting increased efficiency of task performance would lead to lower EMG activity, force error, and SD of force values in the tACS conditions. In contrast to these predictions, these variables were almost identical between the tACS and SHAM conditions over all four time quartiles of the fatiguing contraction. Collectively, these results imply that tACS had no influence on the motor-skill-related components of the fatiguing contraction task or in the associated muscle activation levels in the current study.

### 4.3. Possible Factors Responsible for the Failure of tACS to Improve Fatigue Resistance

The potential contributing factors underlying the absence of a significant influence of tACS on fatigue resistance are difficult to identify. This is due not only to the current investigation being the first on the topic, but also to the fact that substantially fewer motor performance studies in general have involved tACS compared to tDCS. Accordingly, there are fewer studies that have also examined the physiological effects of tACS on human performance relative to tDCS. 

Nonetheless, a few possible factors can be identified and briefly discussed that could have led to the current results based on the available literature on tDCS and tACS, although many are somewhat speculative. First, the most likely explanation is that the parameters of tACS may not have been optimal to improve fatigue resistance, despite their successful use in several tACS motor skill studies. Specifically, the electrode montage, the brain area targeted, the tACS current parameters, and timing (e.g., before vs. during) relative to motor task performance collectively provide an almost infinite combination of possible stimulation paradigms. Nevertheless, subsequent research could start by using tACS paradigms that were successful in other motor performance contexts [26,28,29,30,45]. Second, the single tACS session may not have been sufficient to improve fatigue resistance, and multiple sessions could be needed [10]. However, the vast majority of single-session tDCS motor skill [10] and fatigue studies [16,17,18,19,20,21,22,23] and even the most successful multiple-day tDCS motor studies [14,15,46] were able to demonstrate significant positive effects within one session [10]. Third, it is possible that there could have been ceiling effects due to the study involving only healthy young adults. Accordingly, some studies have shown that the efficacy of tDCS for enhancing motor performance scales with age older adults [47] and with the level of motor impairments displayed by patients with motor disorders. Thus, the more room a participant has for improvement, the more likely tDCS will increase performance [48]. However, similar studies involving muscle fatigue have not been performed. Lastly, the lack of tACS effects could have been due to a combination of the above factors. These possibilities will require extensive research to obtain the resolution to discriminate between these factors. 

On the other hand, the commonly mentioned factors for a lack of positive influences on motor performance in tDCS studies such as low sample sizes, interindividual variability [34,35], and research methodology are likely not applicable to the present study. For instance, the sample size of 26 was greater than the sample size of the majority of tDCS motor skill studies, which appears to be ~13 according to the tables in the review of Buch et al. (2017) [10]. In addition, many tDCS and tACS studies utilized between-subjects designs that have disadvantages compared to the current study’s within-subjects design in regard to statistical power [36] and the much larger anatomical, physiological, and genetic variations between individuals compared to within individuals [34,35]. Furthermore, the futility analysis indicated that it was very unlikely that the lack of significant differences between the *tACS* and SHAM conditions were due to the sample size of the current study. Finally, the fatigue research methodology employed here was consistent with numerous fatigue studies in regard to the use of sustained submaximal isometric contractions in hand muscles to identify the influence of different tasks and interventions as well as the physiological adjustments on fatigue [1,2,3,4,5,6]. 

### 4.4. Limitations

Although the current results were clear in regard to the lack of positive effects of tACS on fatigue resistance using well-established methodology for the investigation of fatigue, the study had several limitations that should be acknowledged. Many of these limitations are interrelated to the factors to blame for the failure of tDCS to increase fatigue resistance as described above. Briefly, the possible limitations of the current study include the following: (1) The electrode montage and tACS parameters employed in the current study were based on studies that successfully improved motor skill as they were the most relevant for motor performance [26,28,29,30,31]; however, other combinations of tACS montages and parameters that also successfully increased motor performance [28] could potentially be more efficacious in mitigating fatigue. (2) It is plausible that tACS could elicit positive effects in other populations that exhibit impairments in motor performance such as older adults [47] and in motor disorders such as multiple sclerosis [49,50,51], as was shown in tDCS studies. Thus, there could have been ceiling effects in the current study, as only young adults were enrolled. (3) There may be more optimal timing paradigms for tACS such as multiple stimulation sessions over consecutive days [10,14,15,46], as used in some tDCS studies. In addition, tACS could be more effective in improving fatigue resistance if applied before compared with during fatiguing contractions, as this timing was successful in some tDCS studies [16,22,23]. (4) A final set of limitations are those related to the general limitations that may be inherent to tDCS and tDCS in some circumstances, such as less current reaching the brain area of interest than predicted [52,53], some participants may not respond as well to the stimulation, and tACS may stimulate peripheral nerves on the scalp [54], which could exert complex effects that could potentially interfere with any positive cortical effects.

## 5. Conclusions

In summary, a single application of tACS delivered concurrently with the performance of a sustained isometric fatigue contraction involving hand muscles did not increase the TTF to a substantial degree compared with the SHAM stimulation. In addition, tACS did not reduce the decrease in the MVC force following the fatiguing contraction. The average EMG, force error, and SD of force increased with time over the course of the fatiguing contractions, but these increases were nearly identical for the tACS and SHAM conditions. Taken together, the current findings offer no evidence that tACS is an effective modality to enhance fatigue resistance, at least in the current task conditions that are typically used to study fatigue during submaximal isometric contractions. Future research should probably focus on the examination of different tACS electrode montages such as the concurrent stimulation of M1 and the cerebellum as well as different sets of tACS parameters that have also been shown to increase motor performance.

## Data Availability

The data presented in the study are available upon request from the corresponding authors.
